# ZnIn_2_S_4_/ZnO Film for High-Efficiency CO_2_ Conversion to Fuel: Photocatalysis by Atomically Disorder-Engineered Heterointerface

**DOI:** 10.1021/acsami.5c08784

**Published:** 2025-08-05

**Authors:** Hossam A. E. Omr, Yu-Ting Wu, You-Heng Siao, Raghunath Putikam, Chen-Kai Chang, Zhe-Wu You, Ming-Chang Lin, Mark W. Horn, Hyeonseok Lee

**Affiliations:** † Department of Photonics, 34874 National Sun Yat-sen University, Kaohsiung 80424, Taiwan; ‡ Department of Applied Chemistry, National Yang-Ming Chiao Tung University, Hsinchu 300093, Taiwan; § Department of Engineering Science and Mechanics, Pennsylvania State University, University Park, Pennsylvania 16802, United States

**Keywords:** ZnO, ZnIn_2_S_4_, CO_2_ photoreduction, heterointerface engineering, Z-scheme charge transfer, film photocatalyst

## Abstract

Light-driven conversion of CO_2_ into small energy-rich molecules effectively addresses both energy demands and reduction in carbon dioxide emissions. However, due to the low efficiency of light absorption and charge carrier separation/transfer, most semiconducting materials have a low conversion activity and poor conversion product selectivity. Herein, ZnIn_2_S_4_ nanosheets are introduced to oxygen vacancy-rich ZnO microrod films for CO_2_ conversion. This heterostructure forms an atomically disordered heterointerface that can play an important role in strengthening the contact between the two crystalline materials and providing an efficient charge transfer pathway. The resulting ZnIn_2_S_4_/ZnO film photocatalyst exhibits superior performance compared to other ZnO-film-based photocatalysts (0.84 and 0.34 μmol·cm^–2^·h^–1^ for CH_4_ and CO, respectively) with ∼90.8% selectivity toward CH_4_ production. The formation of the ZnIn_2_S_4_/ZnO heterojunction film contributes to strengthening the charge carrier generation, separation, and migration through a defect-engineered Z-scheme mechanism. This work highlights the role of disorder-engineered heterointerfaces in film-based heterostructured photocatalysts for optimizing the CO_2_ conversion efficiency.

## Graphical abstract

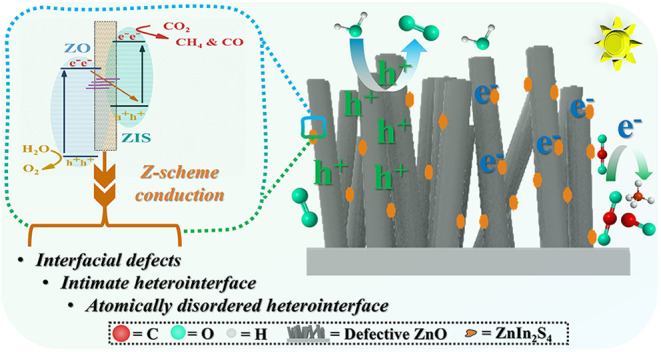

## Introduction

The energy-driven industrialization and unprecedented urbanization of our world have resulted in a dramatic increase in the emission of carbon dioxide (CO_2_) into the atmosphere, which in turn has been linked to severe climate change in recent years.^1^ Sunlight-driven conversion of CO_2_ into valuable fuels by semiconducting materials is a promising technique for addressing environmental changes and the global energy crisis. In 1979, Inoue et al. utilized several semiconductors such as TiO_2_, ZnO, and CdS to reduce CO_2_ to valuable fuels, such as CO, CH_4_, methanol, and ethanol.^2^ Since this innovative work, scientists have reported numerous energetic materials to achieve this photocatalytic reaction, including metal oxides (BiVO_4_, WO_3_, etc.), metal chalcogenides (CdS, CdSe, Zn_*x*_Cd_1–*x*_S, MoS_2_, SnS, SnS_2_, In_2_S_3_, ZnIn_2_S_4_, etc.), metal-free semiconductors (covalent organic framework, conjugated polymers, etc.), and metal–organic frameworks.^3–10^ Among these materials, zinc oxide (ZnO) has been considered a promising n-type semiconductor photocatalyst for CO_2_ conversion due to its excellent properties, such as high chemical stability, favorable band edges for redox reactions, low toxicity, and low-cost synthesis.^11^ Nevertheless, the fast electron–hole recombination, insufficient light absorption due to its wide band gap (∼3.2 eV), and poor photocatalytic CO_2_ conversion performance of ZnO photocatalysts have limited their use in the CO_2_ conversion field.^12,13^ To overcome these shortcomings, various strategies have been investigated to enhance the efficiency of ZnO photocatalysts, such as atomic doping, defect engineering, morphology regulation, single-atom anchoring, and facet engineering.^11,14–17^ In fact, defective ZnO, by introducing vacancies into the crystal structure, has been shown to narrow the band gap and act as photocatalytic active sites for CO_2_ reduction.^15^ Unfortunately, the CO_2_ conversion performance is still limited due to the further aggravated recombination rate of the photogenerated charge carrier.^12^ Heterojunction formation could be a suitable strategy to overcome this obstacle.^18,19^ To date, ZnO has formed a successful heterojunction structure with various semiconductor materials (i.e., CuO, ZnSe, graphene, Bi_2_O_3_, g-C_3_N_4_, metal–organic framework, etc.), achieving excellent photocatalytic activity.^20^

Among the reported heterojunction materials, nanostructured metal sulfides exhibited superior features in photocatalysis.^21^ Thus, heterostructure construction of a ZnO thin film with metal sulfide-based materials is considered a promising candidate for enhancing CO_2_ conversion efficiency. In particular, zinc indium sulfide (ZnIn_2_S_4_) is a ternary metal chalcogenide with a distinct crystal structure that presents many advantages, such as significant visible light absorption due to a favorable band gap (∼2.2 eV), superior CO_2_ adsorption properties, and a larger surface area.^22–25^ ZnIn_2_S_4_ has been reported as a potential candidate for several fields such as photoelectrochemical catalysis, solar cells, photodetectors, light-emitting diodes, light-driven hydrogen production, and photocatalytic CO_2_ reduction.^26–29^ Furthermore, ZnIn_2_S_4_ has been reported as an excellent semiconductor to form junctions with several metal oxides for photocatalytic CO_2_ conversion, including TiO_2_, In_2_O_3_, BiVO_4_, and CuO.^30–33^

Owing to the necessary demand for energy on an industrial scale, scientists have paid great attention to photocatalytic heterostructured thin films due to their numerous advantages, including high catalytic activity, simple handling, long-term recyclability, and precise recollection.^34^ Several ZnO film-based photocatalysts have garnered attention, for example, Iqbal et. al reported a ZnO/ZnTe photocatalytic heterostructure-based film with enhanced light absorption and charge carrier dynamics to achieve CO_2_-to-CH_4_ conversion.^35^ In 2021, Li and coauthors reported the utilization of the plasmonic effect and Z-scheme ZnO/Au/g-C_3_N_4_ film-based photocatalyst for boosting CO_2_ conversion efficiency with 100% selectivity toward CO production (0.0086 μmol·cm^–2^·h^–1^).^36^ Yet, the CO_2_ conversion efficiency over these heterostructured catalysts is still limited for industrial applications due to the poorly regulated interface between the two materials as well as the unsuitable selected materials.

In this study, ZnO microrod films with oxygen vacancies-rich are fabricated by an ethylenediamine (EDA)-assisted hydrothermal method, and then a ZnIn_2_S_4_/ZnO heterostructure is constructed by a facile dispersion dip-coating method, yielding a heterostructure for photocatalytic CO_2_ conversion. The uniqueness of this photocatalytic heterostructure film is characterized by a disordered heterointerface and intimate interfacial contact. The optimum ZnIn_2_S_4_/ZnO photocatalyst exhibits excellent CO_2_ conversion performance (0.84 and 0.34 μmol·cm^–2^·h^–1^ for CH_4_ and CO, respectively) with 90.8% selectivity toward CH_4_. To the best of our knowledge, this performance is the highest CO_2_ conversion rate among ZnO film-based photocatalysts. This remarkable efficiency is ascribed to enhanced light absorption and the formation of a functional heterointerface. In addition, the heterointerface facilitates activation of the Z-scheme charge transfer mechanism at the interface, thereby enhancing the redox ability for efficient and selective CO_2_ conversion. This work demonstrates the vital role of heterointerface engineering in charge carrier migration for high-efficiency CO_2_ conversion by film-based heterostructures.

## Experimental Section

### Chemicals

Zinc foil (99%, 0.4 mm) and ethylenediamine (EDA, 98%) were purchased from Katayama Chemical Inc. Zinc nitrate hexahydrate (Zn­(NO_3_)_2_·6H_2_O, 99.0%) and indium nitrate hydrate (In­(NO_3_)_3_·*x*H_2_O, 99.999%) were purchased from Sigma-Aldrich. Thioacetamide (TAA, 99.0%, ACS reagent) was purchased from Thermo Fisher Scientific. Carbon dioxide (CO_2_, 99.999%) was obtained from Ya Dong Gases CO., Ltd.

### Fabrication of the ZnO Microrod Thin Film

The EDA-assisted hydrothermal method was utilized to fabricate ZnO microrods based on a previously reported work.^36^ In typical procedures, the 2 cm × 2 cm Zn substrate was cleaned with sandpaper (800 grit) and washed three times with ethanol and deionized (DI) water. The cleaned Zn substrate was put into a 100 mL high-pressure Teflon reaction kettle containing a 50 mL mixture of water and ethylenediamine (V/V = 1:1). Then, the autoclave was heated in the muffle furnace at 180 °C for 6, 8, 12, and 16 h. Then, the obtained sample was calcined at 500 °C for 2h with a heating rate of 2 °C/min. Finally, the sample was washed with ethanol and DI water several times and denoted as ZO-*x*, where *x* is the reaction time (in hours) and varies from 6 to 16.

### Synthesis of the ZnIn_2_S_4_ Nanoflakes

The fabrication process of ZnIn_2_S_4_ nanoflakes is achieved by the hydrothermal method.^23^ Typically, 1 mmol of Zn­(NO_3_)_2_·6H_2_O (0.297 g), 2 mmol of In­(NO_3_)_3_·*x*H_2_O (0.762 g), and 2 mmol of thioacetamide (TAA, 0.15 g) were dissolved in 70 mL of deionized water and transferred to the 100 mL high-pressure Teflon reaction kettle and heated in the muffle furnace at 120 °C for 10h. The collected product was washed with deionized water several times, dried at 60 °C, and the final product was marked as ZIS.

### Fabrication of the ZnIn_2_S_4_/ZnO Nanostructure Catalysts

The decoration of ZnIn_2_S_4_ on the ZnO microrods was achieved via the dispersion dip-coating method, as shown in Scheme 1. In detailed procedures, the as-prepared ZnO film (ZO-*x*) was immersed in the 0.25 g/L ZnIn_2_S_4_ ethanolic dispersion for 1h. Then, the as-fabricated composite was dried in a drying oven at 70 °C overnight and denoted as ZIS/ZO-*x*, where *x is* the reaction time of the prepared ZnO.

**Scheme 1:**
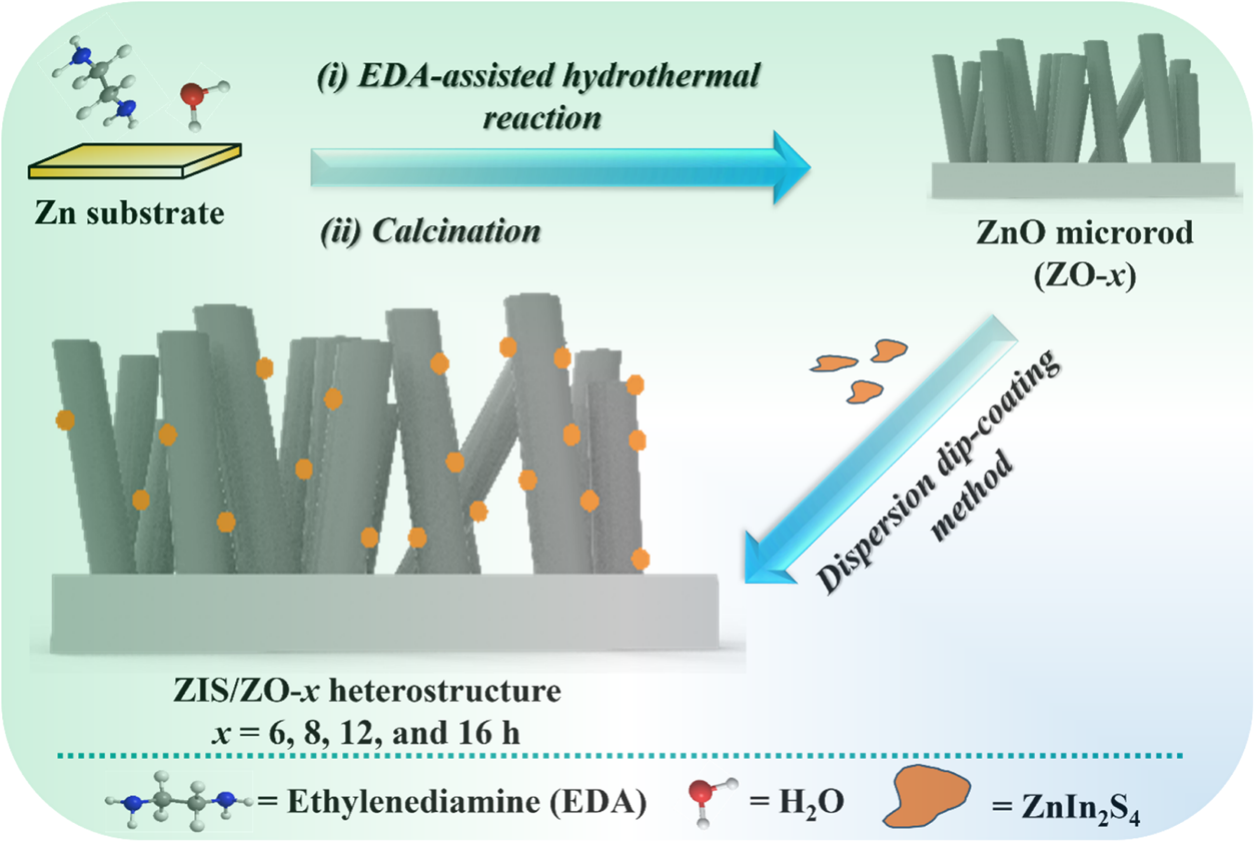
Schematic Diagram for the Fabrication of ZIS/ZO-*x* Heterostructures

### Characterization

Raman spectra were recorded on a 3D Nanometer Scale Raman PL Microspectrometer (Tokyo Instruments, INC.). An X-ray diffractometer (XRD, Rigaku MiniFlex) was used to determine the crystal structure. A field-emission scanning electron microscope (FE-SEM, Zeiss Gemini 450 microscope, with an acceleration voltage of 30 kV under a vacuum pressure of 10^–10^ Torr) was used to investigate the surface morphologies of the samples. Transmission electron microscopy (TEM, JEOL JEM-3010, 300 kV high-energy electron) was utilized to identify the nanostructure of materials. UV–vis diffuse reflectance spectroscopy (UV–vis DRS Shimadzu, UV-2600i) was employed to analyze the optical properties. The elemental composition of the materials was identified by X-ray photoelectron spectroscopy (XPS, JEOL, JAMP-9500F). Ultraviolet photoelectron spectroscopy (UPS, Prevac, XPS/UPS system) was employed to measure the work function. The electrochemical properties were measured by an electrochemical station (CHI-660E, CH Instruments, Inc.). A solar simulator equipped with AM1.5G (LCS-100, 94001 A, Newport) was used as a light source for the photocatalytic reaction. Gas chromatography (GC, Shimadzu, GC-2030 Nexis) was utilized to determine the concentration of gaseous species. The GC was equipped with a standard Split Liner (SPL) at a temperature of 150 °C and a barrier discharge ionization detector (BID-2030) at a temperature of 300 °C. The column was supplied with a micropacked column (Shinwa Chemical Industries Ltd.).

### Photocatalytic CO_2_ Conversion

The photocatalytic experiments were performed in a 25 mL two-valve reactor made of stainless steel and equipped with a quartz window on the top. Before the reaction was started, the reactor was filled with CO_2_ gas, vacuumed, and purged several times. Subsequently, high-purity CO_2_ gas (99.999%) was mixed with water through a water bubbler containing 9 mL of deionized water and injected into the reactor until it equilibrated with atmospheric pressure. The reactor was put under the solar simulator for illumination for 3 h. Finally, an airtight syringe was used to extract the product species for injection into the GC instrument to measure the CO_2_ conversion efficiency.

## Results and Discussion

The structural properties of the ZnO, ZIS, and ZIS/ZnO-*x* photocatalysts were analyzed by X-ray diffraction (XRD) and Raman spectroscopy techniques. As shown in Figures 1a and S1, the diffraction pattern of ZO-*x* catalysts displays a set of diffraction peaks at 2θ = 31.86, 34.6, 36.2, 47.63, 56.69, 62.8, 67.98, and 68.95°, which are assigned to the (100), (002), (101), (102), (110), (103), (200), and (112) crystal planes of ZnO, respectively, confirming the hexagonal lattice structure of ZnO (JCPDS # 89-0510, *a* = 3.2498 Å, *b* = 3.2498 Å, and *c* = 5.2066 Å) for all fabricated ZO catalysts.^26,37^ Additionally, the XRD pattern of ZIS material demonstrated that ZIS grown in the cubic crystal structure of ZnIn_2_S_4_ as proved by the presence of signals at 2θ = 27.96, 33.8, 44.16, and 48.43° which matches with (311), (400), (511), and (440) diffraction planes of ZnIn_2_S_4_ (JCPDS # 48-1778), respectively.^38^ After the decoration of the ZO-*x* film with ZIS materials, the diffraction pattern of all nanocomposites has the characteristic signals only for ZnO materials. This result could be ascribed to the small amount of ZIS materials deposited on the surface of the ZO film.^39^ Figure 1b shows the Raman spectra of the ZO-12, ZIS, and ZIS/ZO-12 photocatalysts. The group theory indicated that the Raman spectra of wurtzite ZnO have a set of optical phonon modes (such as A1 and E2), and the A1 mode splits into longitudinal optical (LO) and transverse optical (TO) components.^40^ ZO-12 has four distinct signals at Raman shift of 328, 378, 433, and 572 cm^–1^, which are indexed to (E_2_^high^-E_2_^low^), A_1_(TO), optical phonon (E_2_^high^), and 1LO modes, respectively, suggesting the successful formation of hexagonal ZnO.^41–43^ ZIS displays the LO_1_, TO_2_, and LO_2_ modes of ZnIn_2_S_4_ at 241, 301, and 350 cm^–1^, respectively.^44^ For the heterostructured ZIS/ZO-12 catalyst, the peak broadness with decreased intensity, together with the slight wavenumber redshift for the signal assigned to the 1LO mode (the inset of Figure 1b), indicates the existence of structural oxygen disorder in the ZnO lattice after heterostructure formation.^45–47^

**Figure 1:**
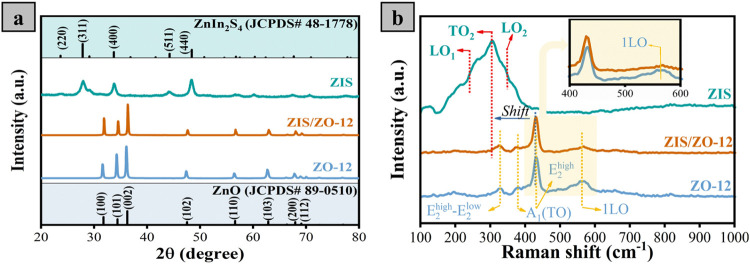
XRD patterns (a) and Raman spectra (b) for the ZO-12, ZIS, and ZIS/ZO-12 catalysts.

To analyze the morphological structure of the synthesized photocatalysts, field-emission scanning electron microscopy (FE-SEM), transmission electron microscopy (TEM), and high-resolution TEM (HR-TEM) analyses were performed. Figures 2a–c, S2, and S3 show the surface morphologies of the ZO-*x* catalysts prepared at different reaction times. Figures 2a–c and S2 show that ZO-*x* (*x* = 6, 8, and 12 h) catalysts exhibit a microrod structure with an approximate film thickness of 8.45, 13.50, and 15.17 μm, respectively, as deduced from cross-sectional SEM images. An increase in the reaction time (i.e., *x* = 16 h) drastically changes the unique microrod morphology (Figure S3). Figure 2d shows the FE-SEM image of ZIS/ZO-12, suggesting that ZIS is successfully deposited on the surfaces of ZO microrods.

**Figure 2:**
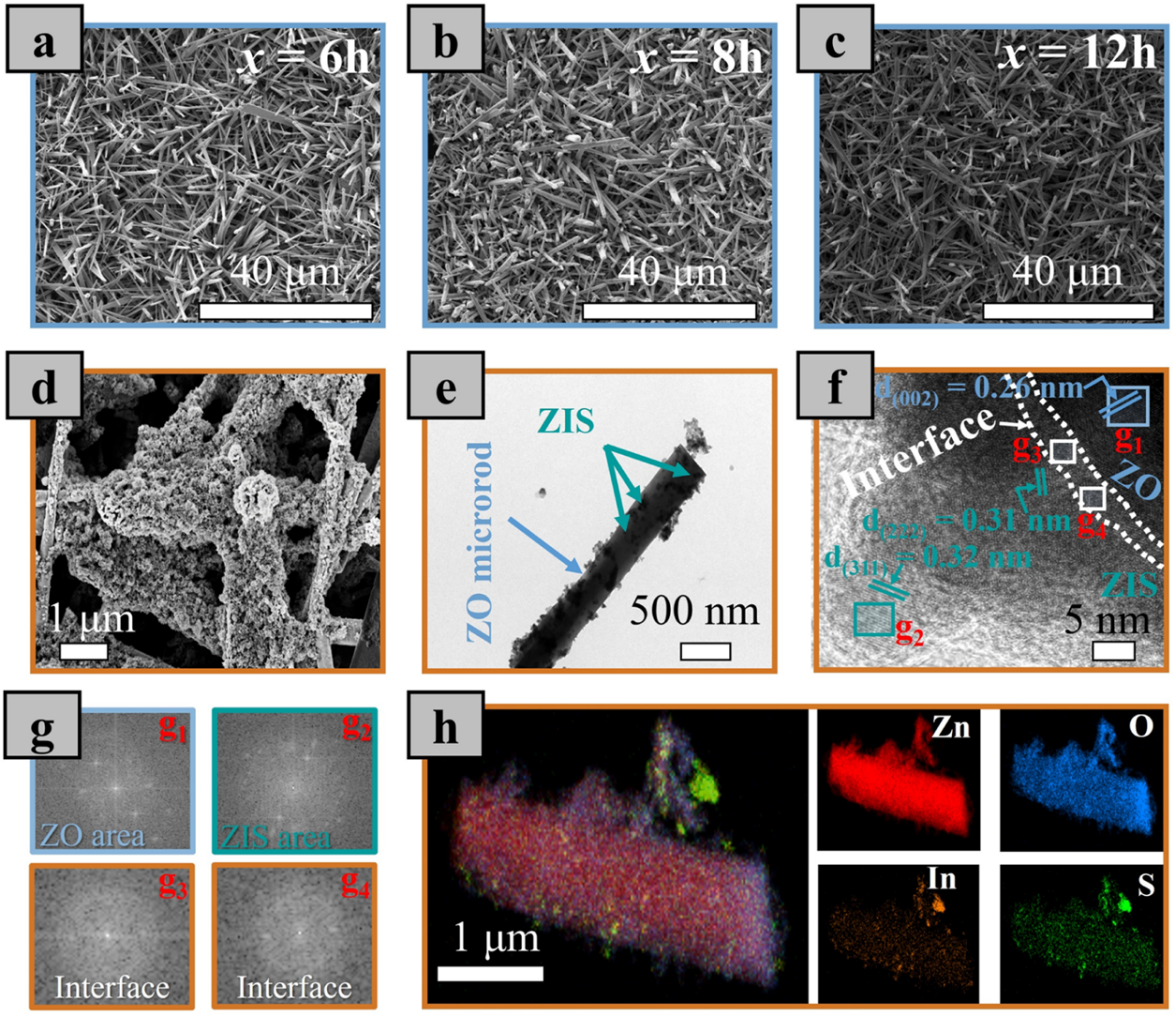
FE-SEM images of (a) ZO-6, (b) ZO-8, (c) ZO-12, and (d) ZIS/ZO-12. TEM (e) and HR-TEM (f, g) images show the corresponding FFT patterns for the marked square areas of ZIZ/ZO-12. (h) HAADF-STEM and corresponding EDS elemental map images for the ZIS/ZO-12 catalyst.

To further investigate the nanostructure of the catalysts, TEM measurements for ZIS and ZIS/ZO-12 are implemented in Figures S4 and 2e, respectively. ZIS shows a nanosheet morphological structure, possessing (311) lattice fringes with 0.32 nm spacing as displayed in the HR-TEM image (Figure S4a,b), which indicates the growth of ZnIn_2_S_4_ nanosheets with the cubic crystal structure. In addition, the SAED pattern demonstrates the polycrystalline nature of the ZnIn_2_S_4_ material (Figure S4c). Figure 2e shows the TEM image of ZIS/ZO-12, revealing that ZO-12 is uniformly decorated with ZIS nanoflakes, which suggests the delicate synthesis of the heterostructured photocatalyst. Moreover, the HR-TEM images demonstrate the existence of the lattice fringes with distances of 0.31, 0.32, and 0.28 nm, corresponding to (222) and (311) crystal planes of ZnIn_2_S_4_, and (002) crystal plane of ZnO, respectively. Interestingly, the presence of crystal disorder is identified at the ZIS/ZO-12 heterointerface. The disordered heterointerface is further confirmed by Fast Fourier Transform (FFT) analysis in Figure 2g. The ZO and ZIS areas are characterized by a high crystalline phase, while the FFT images at the heterointerface demonstrate amorphous features at the ZIS/ZO-12 catalyst. This could be ascribed to the fact that the heterostructure formation results in a disordered atomic arrangement near the interface of the two materials, as reported by other works,^48,49^ and this is beneficial to form an excellent intimate contact.^50^ The HAADF-STEM and EDS elemental mapping images of ZIS/ZO-12 in Figure 2h confirm that ZnIn_2_S_4_ elements are homogeneously distributed on the ZnO material, proving uniform formation of ZnIn_2_S_4_ /ZnO nanocomposites. Moreover, the elemental composition of the heterointerfaces was investigated by a STEM-EDX line profile (Figure S5). Obviously, the interface is mainly composed of ZnO variant (i.e., Zn and O elements) with minor In and S atoms. The existence of this atomically disordered heterointerface was further investigated by density functional theory (DFT) simulations, as shown in Figure S6. The bond length variations at the interface of the heterostructured catalyst compared to pure ZIS and ZnO are indicative of the structural disorder at the interface.^51^ It is hypothesized that the lattice mismatch and atomic dislocations resulted in the atomically disordered heterointerface, as reported by other works.^51–53^

To study the chemical composition and bonding configuration of ZO-*x*, ZIS, and ZIS/ZO-12, X-ray photoelectron spectroscopy (XPS) measurements were implemented. Figure S7 shows that the ZO-*x* catalyst has two characteristic signals for the Zn and the O elements. For HR-XPS Zn 2p, the binding energies at 1020 and 1043.2 eV are related to the characteristic Zn 2p_3/2_ and Zn 2p_1/2_ of ZnO materials, respectively.^54^ No shift is observed in Zn 2p with increasing reaction time, which indicates that prolonging the reaction time does not influence the oxidation state of Zn. The HR-XPS of O 1s for ZnO materials has two signature peaks at 529.1 and 530.3 eV that originate from the oxygen lattice (O_L_) and the oxygen vacancies (O_V_), respectively.^36^ The O_V_ area is gradually increased by increasing the reaction time and reaches a maximum at *x* = 12 h, as listed in Table S1, suggesting that the ZO-12 catalyst has the highest defective structure among all ZO-*x* catalysts.

Figure 3a displays the two characteristic signals for Zn 2p_3/2_ and Zn 2p_1/2_ for ZnO-12, ZIS, and ZIS/ZO-12. Importantly, the peaks corresponding to O 1s for ZIS/ZO-12 are slightly shifted toward high binding energy, suggesting electron loss after heterostructure formation. In addition, the concentration of O_V_ is decreased after heterojunction formation in the ZIS/ZO-12 catalyst (Table S1), indicating the suppression of defects by ZIS decoration. It is speculated that this is attributed to the passivation effect while the formation of the ZnIn_2_S_4_/ZnO heterostructure, which is signified by the presence of the amorphous layer in Figure 2f–g.^55^ This also indicates that deposited ZnIn_2_S_4_ is strongly coupled with ZnO at the interface of the heterostructured catalyst with a reduced content of interfacial defects. Figure 3c shows the two distinct peaks in the HR-XPS In 3d spectrum for ZIS and ZIS/ZO-12. The two signature peaks of ZIS/ZO-12 are negatively shifted compared with those of ZIS, demonstrating the electron-rich In sites after junction formation. Similarly, the ZIS/ZO-12 represents the same trend for HR-XPS of S 2p, implying electron transfer from the ZO to ZIS after heterostructure formation, as shown in Figure 3d. The decreased peak intensity for S 2p, as in the case of O 1s in ZIS/ZO-12, is affected by a strong interaction between ZnIn_2_S_4_ and ZnO materials, probably due to the passivation effect.

**Figure 3:**
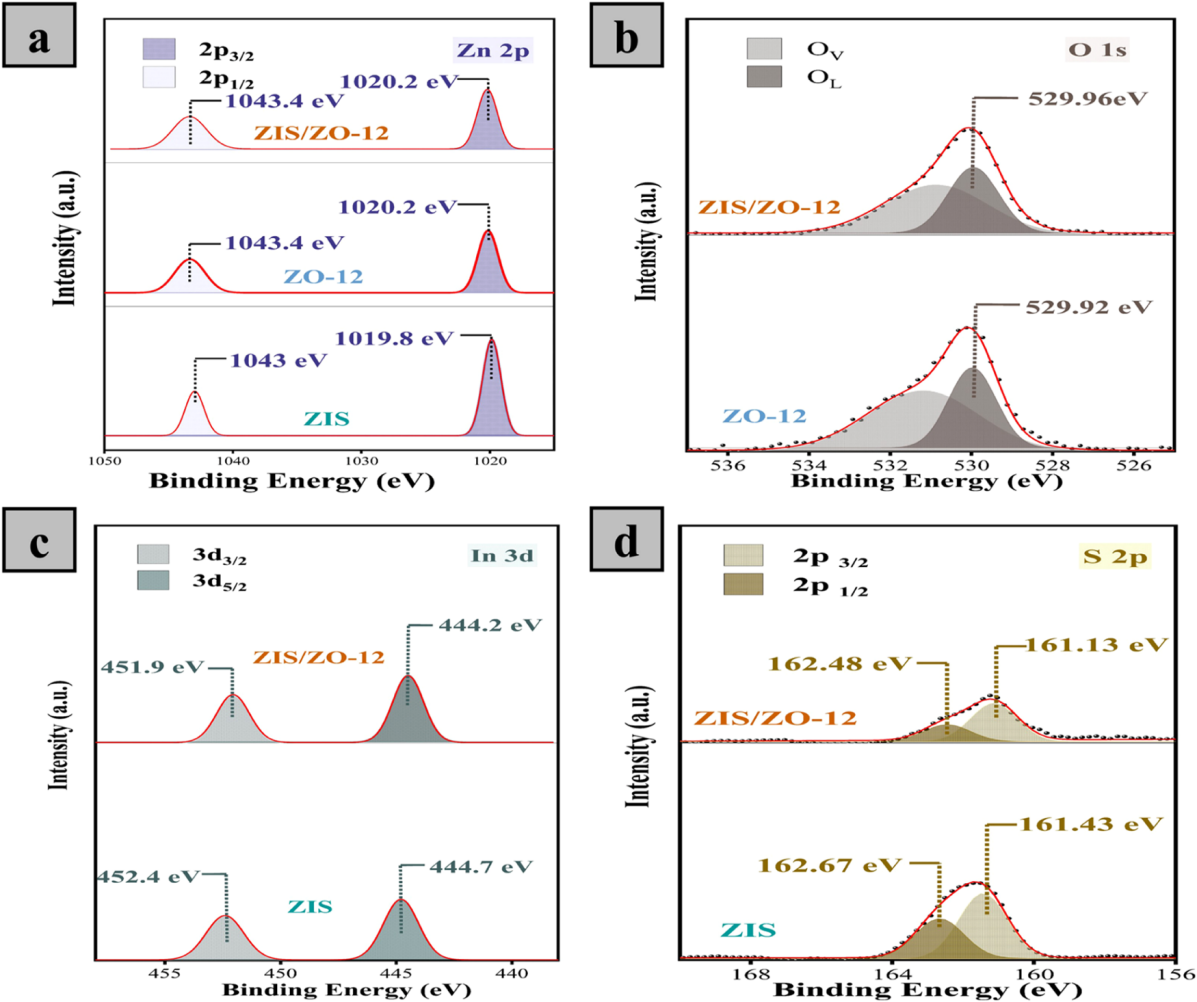
(a) HR-XPS spectra of Zn 2p for ZO-12, ZIS, and ZIS/ZO-12. (b) HR-XPS of the O 1s for ZO-12 and ZIS/ZO-12. HR-XPS spectra of (c) In 3d and (d) S 2p for ZIS and ZIS/ZO-12.

The CO_2_ conversion performance was evaluated for the ZO-*x* catalysts, as shown in Figures 4a and S8. All ZO-x catalysts exhibit only CO_2_-to-CH_4_ conversion, and ZO-12 produces the highest conversion rate of 0.60 μmol·cm^–2^·h^–1^, probably due to the well-microstructured ZnO material with enlarged reaction sites by increased film thickness (Figure S2). After the formation of the ZnIn_2_S_4_/ZnO heterostructure, all ZIS/ZO-*x* photocatalysts exhibit a higher or comparable CH_4_ production rate in comparison with that of ZO-12 catalysts, and the heterostructured samples produce CO additionally in Figure 4a. This might be attributed to the fact that the ZnIn_2_S_4_ surface is favorable for CO and CH_4_ desorption during photocatalysis, as reported by other works.^30,56^ Our measured result of ZIS also corresponds to this, which shows CO and CH_4_ production rates of 0.29 and 0.24 μmol·cm^–2^·h^–1^, respectively, as presented in Figure 4a. Importantly, the ZIS/ZO-12 composite reaches an optimum CO_2_ conversion with production rates of 0.84 and 0.34 μmol·cm^–2^·h^–1^ for CH_4_ and CO, respectively, with 90.8% selectivity toward CH_4_ production. To the best of our knowledge, this performance is the highest CH_4_ production rate by ZnO film-based photocatalysts as listed in Table S2. Moreover, ZIS/ZO-12 produces almost identical CH_4_ and CO production rates without remarkable degradation in the material after the four-time measurements, indicating excellent recyclability as displayed in Figure 4b. In addition, the production yield of the gaseous species (i.e., CO and CH_4_) increased gradually with illumination time and remained constant up to 18 h, as shown in Figure S9a. The existence of a new signal at 169.1 eV in HR-XPS of S 2p (Figure S9b) demonstrates the oxidation of sulfide after 18 h of continuous photocatalysis, which deactivates the photocatalyst. To assess the reliability of the production rate measurement, control experiments are conducted under various reaction conditions, such as without light illumination, without water, and with argon gas instead of CO_2_, as shown in Figure 4c. No CH_4_ or CO species are detected among all control tests, demonstrating that CO and CH_4_ are produced from light-driven CO_2_ reduction in the presence of water. In addition, the photocatalytic reaction is performed with the isotopic ^13^CO_2_, as shown in the inset of Figure 4c, to further confirm the origin of the produced gaseous species. The two signals that are recorded at *m*/*z* = 17 and 29 are related to ^13^CH_4_ and ^13^CO, respectively, indicating that CO_2_ is the main carbon source for the formation of CH_4_ and CO by the fabricated photocatalyst. To confirm the stability of the catalyst, XRD measurements for the catalyst before and after photocatalysis are performed and plotted in Figure 4d. No significant peak shift in the XRD pattern after four cycles of photocatalysis, indicating the high structural stability of the catalyst.

**Figure 4:**
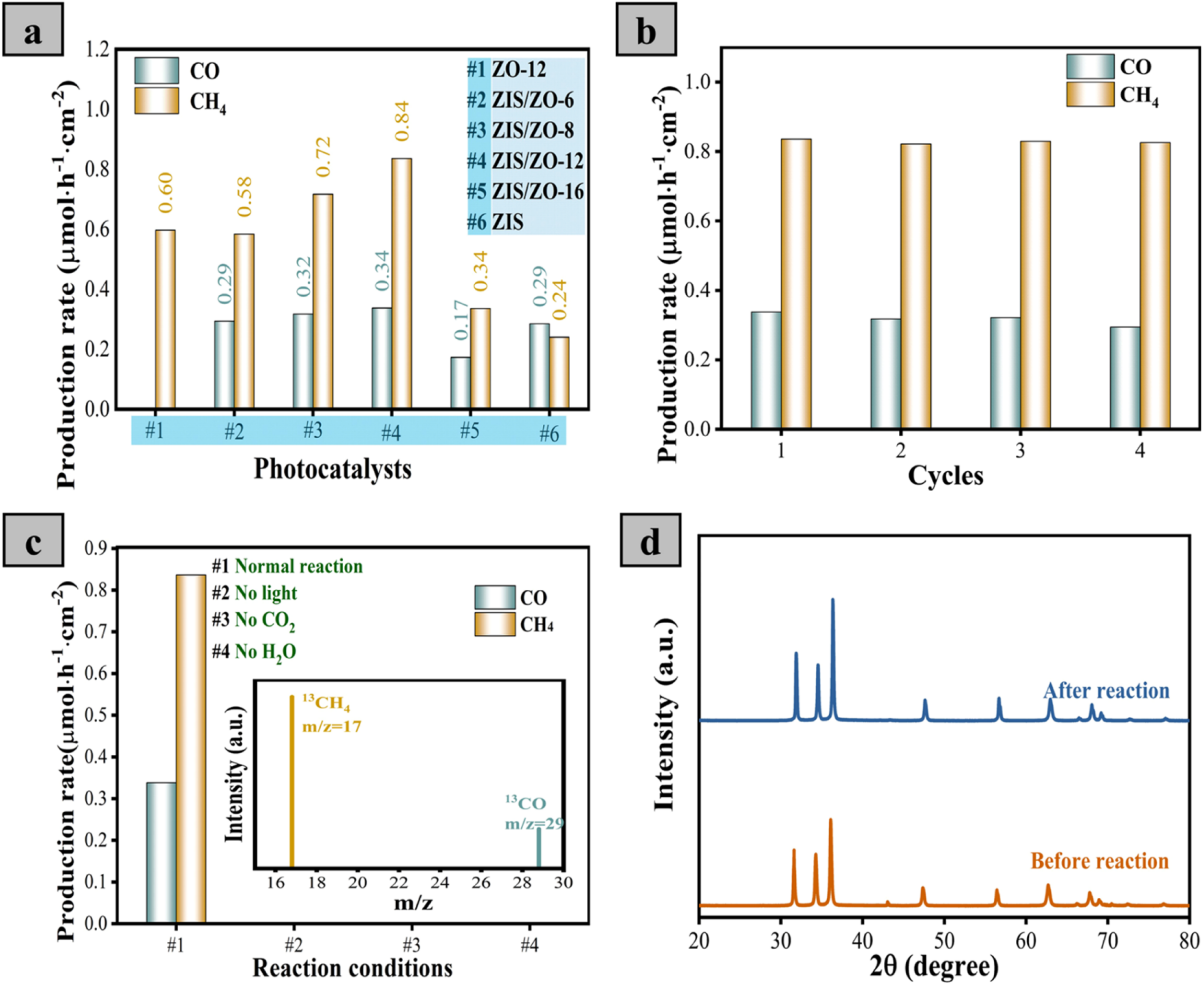
(a) CO and CH_4_ Production rates measured by ZO-12, ZIS/ZO-*x.* (b) Recyclability experiments and (c) control tests by ZIS/ZO-12 photocatalyst (the inset is GC-MS analysis using ^13^CO_2_ for photocatalytic conversion by ZIS/ZO-12 catalyst). (d) XRD patterns of ZIS/ZO-12 before and after four photocatalytic CO_2_ conversions.

To analyze the high efficiency and selectivity of the CO_2_ conversion performance by our fabricated photocatalysts, the optical and electrochemical properties were studied by UV–vis diffuse reflectance spectroscopy (UV–vis DRS), photoluminescence spectroscopy (PL), time-resolved PL (TR-PL), and electrochemical impedance (EIS) techniques. Figure 5a displays the absorbances of ZO-12, ZIS/ZO-12, and ZIS materials. ZO-12 shows strong absorbance only in the UV region, and this could be ascribed to the wider band gap of ZnO as demonstrated from the Tauc plot (E_g_ = 3.17 eV) in the inset of Figures 5a andS10 while the light absorption spectra of ZIS extends to the visible region owing to the narrow band gap of ZnIn_2_S_4_ material (E_g_ = 2.2 eV). After heterostructuring of ZO-12 with ZIS, a slight enhancement in visible light absorption is observed, as displayed in Figure 5a, suggesting the enhanced CO_2_ conversion efficiency by the fabricated composites as presented in Figure 4a. ZnO material exhibits a strong emission band in the wavelength range of 370–500 nm.^57,58^ The photoluminescence of the ZO-*x* catalysts is recorded, as shown in Figure 5b. Notably, the ZO-12 has the lowest emission peak intensity among all catalysts, indicating a strong quenching effect that is affected by a higher degree of localized energy states by oxygen vacancies,^59^ as shown in Figure 3 and Table S1. It can be observed that the PL intensity of the ZO-12 catalyst gradually reduced after the construction of a heterostructure with ZIS (Figures 5c and S11), suggesting the declined recombination rate as a result of charge transfer at the interface between the two materials.^60,61^ Notably, the ZIS/ZO-12 catalyst has the highest interfacial charge transfer among all heterostructured photocatalysts (Figure S8). This could be ascribed to the intimate contact via a structurally disordered amorphous boundary at the heterointerface, as in Figure 2f, which helps to facilitate the photogenerated charge transfer.^62,63^ In addition, several works have reported that interfacial passivation is beneficial in the enhancement of interfacial charge transportation further.^55,64^ The shortened carrier lifetime of the heterostructured catalyst (1.71 ns) compared to the ZO-12 catalyst (2.88 ns) in Figure S12 further confirms the effective interfacial charge transportation.^60^ Thereby, the significant enhancement of charge carrier migration that can boost the CO_2_ conversion is available through our ZIS/ZO-12 catalyst. To investigate the characteristics of charge transfer between adsorbed CO_2_ and the catalyst, EIS measurements were conducted in a KHCO_3_ electrolyte saturated with CO_2_. ZO-12 has the lowest semicircle diameter of the Nyquist plot as shown in the inset of Figure 5d, demonstrating the low charge transfer resistance of this catalyst at the interface between the adsorbed CO_2_ species and the photocatalyst. Importantly, the charge transfer resistance is further declined after junction formation with ZIS, as presented in Figure 5d, meaning that the interface between CO_2_ and the catalyst becomes more conductive in the heterostructured catalyst, which is another beneficial feature for a high rate of CO_2_ conversion. All of the above systematic measurements support the highest CO_2_ conversion performance by ZIS/ZO-12.

**Figure 5:**
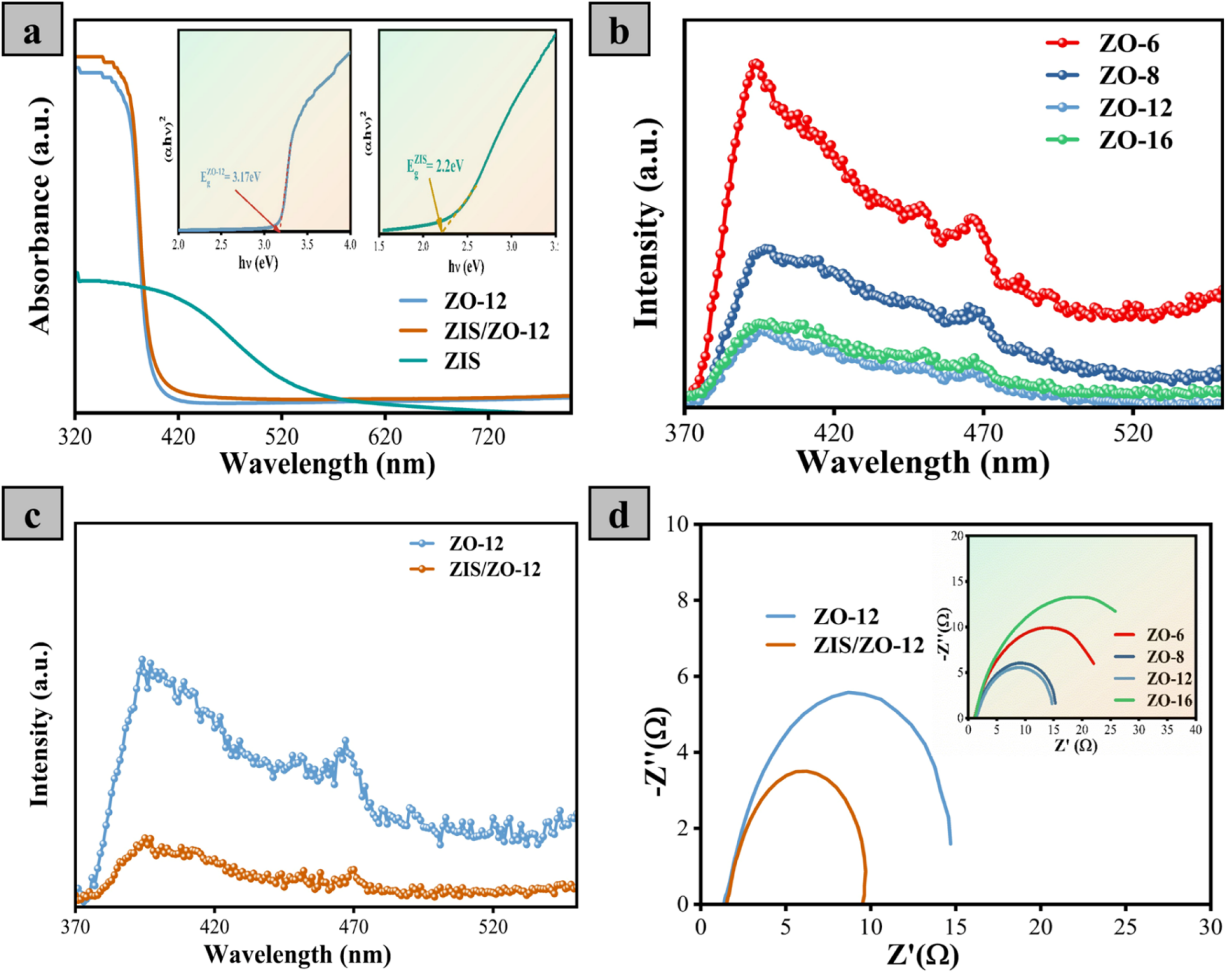
(a) UV–vis spectra for ZO-12, ZIS, and ZIS/ZO-12 (the inset is a Tauc plot for the ZIS and ZO-12 catalysts). PL spectra for (b) ZO-*x* and (c) ZO-12 and ZIS/ZO-12 heterostructured catalyst. (d) EIS spectra for ZO-12, ZIS/ZO-12 (the inset is the EIS spectra for ZO-*x* materials).

The charge transfer mechanism at the interface of the two materials in the fabricated heterostructured catalyst was investigated by UV-photoelectron spectroscopy (UPS) and valence band XPS techniques in combination with UV–vis spectroscopy. Figure 6a,b shows the UPS results for ZO-12 and ZIS. Based on the literature,^61,65^ the work function (ϕ) can be extracted from the UPS spectrum by the equation ϕ = hν – (E_cutoff_ – E_f_), where hν, E_cutoff_, and E_f_ are the excitation energy (i.e., 21.22 eV for He I), secondary cutoff energy (eV), and Fermi level (eV), respectively. Herein, the calculated work functions are 4.45 and 4.58 eV for ZO-12 and ZIS, respectively.^66,67^ The larger work function of ZIS further proved the electron transfer from the ZO microrod to ZIS nanosheet to form a heterojunction under thermal equilibrium conditions, which corresponds to the analysis by XPS in Figure 3. In addition, the energy levels of the valence band maximum (E_VBM_) for ZO and ZIS are investigated by VB-XPS in Figure 6c,6d. The extracted values of E_VBM_ for ZO and ZIS are 2.79 and 0.96 eV, which can be converted to 2.72 and 0.89 V (vs NHE), respectively. Subsequently, the locations of the conduction band minimum (E_CBM_) are estimated to be −0.45 and −1.31 V for ZO-12 and ZIS, respectively, by considering their optical band gaps. The band energy structures of ZO-12 and ZIS depict all of this information in Figure 7a. Considering all the values, ZO and ZIS form a typical staggered band energy configuration for the junction formation. The constructed band structure of ZO is favorably matched to the reduction potential of CO_2_ to CH_4_, while ZIS has well-aligned E_CBM_ and E_VBM_ with the redox potentials of CO_2_/CO, CO_2_ /CH_4_, and water oxidation. This would be the primary reason for the selective production of our catalysts in Figure 4. Figure 7b describes the proposed charge transfer mechanism for the ZIS/ZO heterostructured catalyst. After illuminating the catalyst, the photogenerated electrons are excited from VB to CB of ZIS and ZO, absorbing light energy at longer wavelengths due to a smaller band gap of ZIS. Subsequently, the excited electrons in the CB of ZO migrate to the VB of ZIS despite the presence of an unfavorable internal electric field because of the presence of interfacial defects via the intimate contact at the ZIS/ZO heterointerface.^68,69^ This activates Z-scheme charge transfer, accumulating electrons in the CB of ZIS that could achieve the reduction of CO_2_ to solar fuels, while the holes in the VB of ZO can achieve the water oxidation reaction, combined with the innate excellent CO desorption capability of the ZnIn_2_S_4_ surface during the photocatalytic reaction.^30,56^ This proposed mechanism could enhance the charge carrier transportation and maximize the redox ability of the photogenerated electrons and holes,^70^ achieving excellent CO_2_ conversion efficiency as shown in Figure 4. The functional role of the disordered interface was examined by conducting a charge density difference (CDD) simulation. Figure 7c reveals that the ZnIn_2_S_4_/ZnO catalyst has an efficient interfacial charge migration and highly redistributed charge density at the interface, with accumulated electrons on the ZnIn_2_S_4_ surface in the heterostructured catalyst. Also, the adsorption–desorption properties of the reactants/products of the photocatalytic CO_2_ conversion were calculated for the photocatalysts, as presented in Figure 7d,e. The heterostructured catalyst has more negative adsorption energies for the reactants (*E*_ads_^CO_2_^ = −0.47 eV *and E*_ads_^H_2_O^ = −1.17 eV) compared to the pure catalysts (Figure 7d), demonstrating the strong adsorption affinity of ZnIn_2_S_4_/ZnO toward CO_2_ and H_2_O, which is a critical step for activating the reactant molecules for a high-efficiency CO_2_ conversion process. In addition, the CO and CH_4_ desorption energies were simulated as presented in Figure 7e. It is demonstrated that the desorption of CO intermediates on the ZnO surface is not favorable as a result of the strongly adsorbed CO on the surface (*E*_des_^CO^ = +1.12 eV), suggesting further reduction to produce CH_4_ as a product. In sharp contrast, the desorption of CO and CH_4_ on the heterostructured catalyst is more favorable, as demonstrated by the lower energy values of the ZnIn_2_S_4_/ZnO catalyst (*E*_des_^CO^ = +0.40 eV and *E*_des_^CH_4_^ = +0.38). These results suggest that the coupling of ZIS with ZnO helped in enhancing the CO_2_ conversion into CH_4_ and CO as major and minor products, respectively.

**Figure 6:**
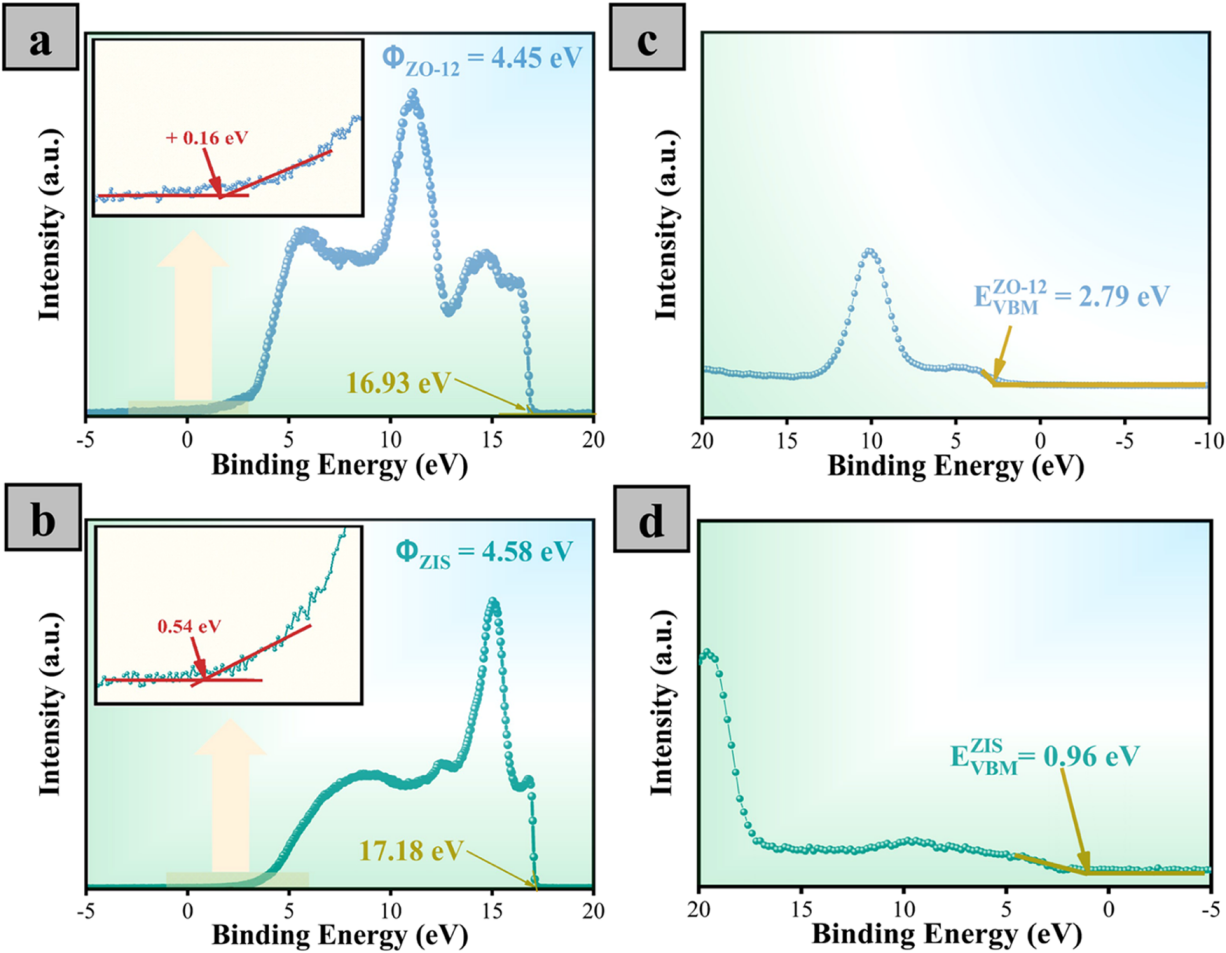
UPS spectra of (a) ZO-12 and (b) ZIS. VB-XPS spectra of (c) ZO-12 and (d) ZIS.

**Figure 7:**
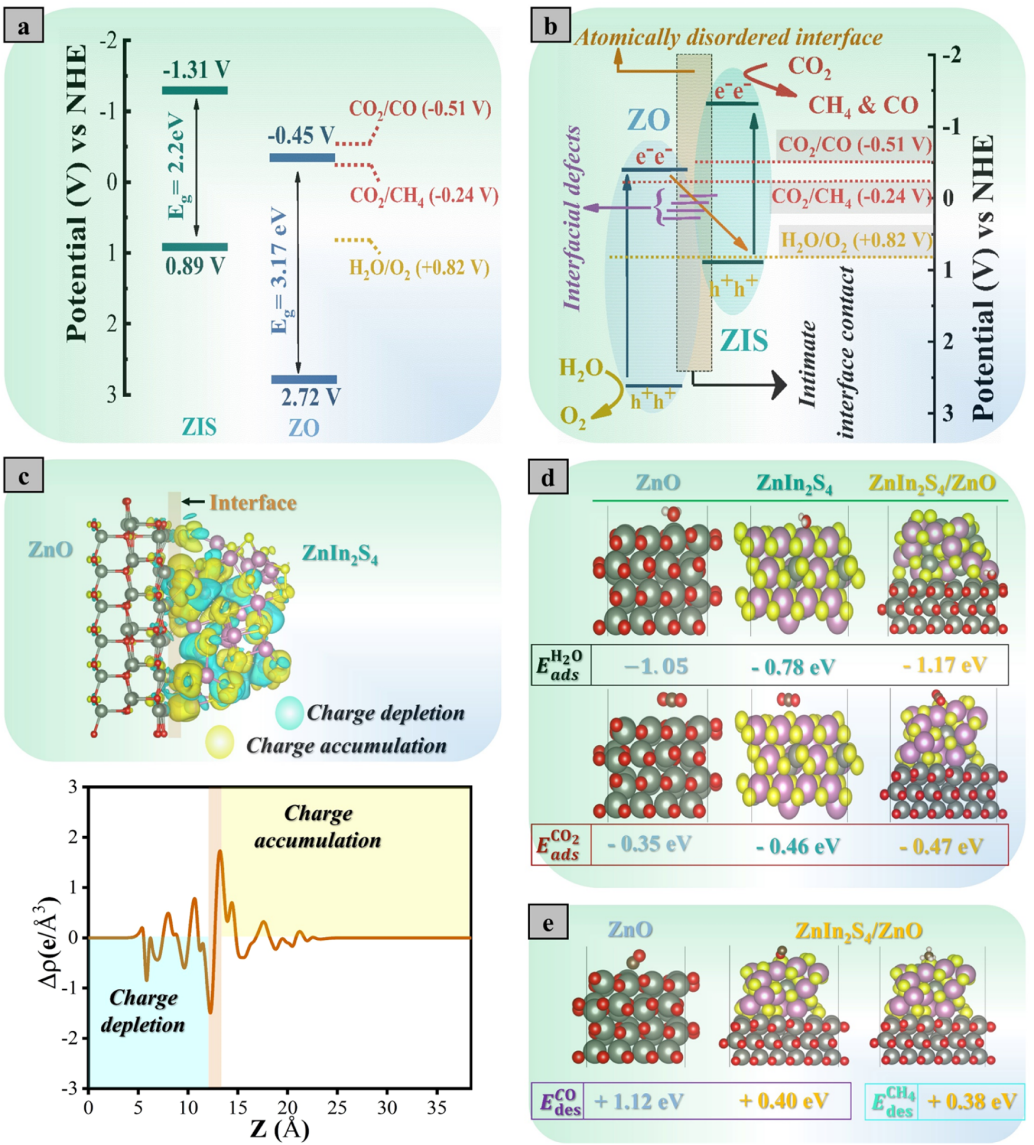
(a) Band energy diagram for ZIS/ZO before junction formation. (b) Proposed charge transfer mechanism by the heterostructured photocatalyst. (c) Simulated charge density difference and corresponding calculated planar-averaged charge density distribution for ZnIn_2_S_4_/ZnO heterostructured catalyst. (d) The simulated structure of CO_2_ and H_2_O adsorbed on ZnO, ZnIn_2_S_4_, and ZnIn_2_S_4_/ZnO catalysts. (e) Simulated structure of CO and CH_4_ on ZnO and ZnIn_2_S_4_/ZnO catalysts.

## Conclusions

In conclusion, ZnIn_2_S_4_/ZnO heterostructured film photocatalysts were synthesized by dispersion dip-coating of ZnIn_2_S_4_ onto disorder-engineered ZnO nanorod films, and they showed improved photocatalytic CO_2_ conversion efficiency and selectivity. The ZnIn_2_S_4_/ZnO heterostructures are characterized by a unique amorphous layer at the heterointerface that can provide localized energy states and a passivation effect for charge transfer through the interface. The optimal heterostructured film photocatalyst achieved excellent performance and high stability on CO_2_ conversion with CH_4_ and CO evolution rates of 0.84 and 0.34 μmol·cm^–2^·h^–1^, respectively. The improved performance is mainly attributed to (i) enhanced light absorption ability due to the incorporation of ZnIn_2_S_4_ with a small band gap, (ii) the promoted separation and migration of the photogenerated charge carrier by the atomically disordered heterointerface, and (iii) the spatial separation of oxidative reductive active sites through Z-scheme conduction with maximum redox ability for the photogenerated electrons and holes. This work demonstrates how disorder-engineering of the heterointerface in film-based heterostructured photocatalysts can enable stable and high-efficiency CO_2_ conversion.

## Supplementary Material



## Notes

Additional details on the simulation method, XRD, FE-SEM, TEM, SAED, HAADF-STEM, and corresponding EDX mapping, DFT simulation, XPS, TR-PL, CO_2_ performance of the catalysts, and comparison of the photocatalytic activity (PDF)
