# Supplementary material for: ZnIn2S4/ZnO Film for High-Efficiency CO2 Conversion to Fuel: Photocatalysis by Atomically Disorder-Engineered Heterointerface
Source: ACS Appl Mater Interfaces. 2025 Aug 5;17(33):47625–36. doi: 10.1021/acsami.5c08784 (PMC12371694; doi:10.1021/acsami.5c08784)
Supplement: Supplementary file 1 [file am5c08784_si_001.pdf]

Supporting Information

$ZnIn_2S_4/ZnO$ film for high-efficiency CO_2 conversion to fuel: photocatalysis by atomically disorder-engineered heterointerface.

Hossam A. E. Omr [□,‡], *Yu-Ting Wu* [□,‡], *You-Heng Siao*[□], *Raghunath Putikam* [ʃ], *Chen-Kai Chang*[□], *Zhe-Wu You*[□], *Ming-Chang Lin* [ʃ], *Mark W. Horn*[§], *and Hyeonseok Lee* [□,*]

[□] Department of Photonics, National Sun Yat-sen University, Kaohsiung 80424, Taiwan

[ʃ] Department of Applied Chemistry, National Yang-Ming Chiao Tung University, Hsinchu 300093, Taiwan

[§] Department of Engineering Science and Mechanics, Pennsylvania State University, University Park, PA 16802, USA

‡ These two authors equally contributed to this work

* Correspondence: hslee611@mail.nsysu.edu.tw

Section I. Simulation Method

The first-principles calculations were achieved using the Vienna ab initio simulation package (VASP) [1, 2]. The projector augmented wave (PAW) method was used to describe the ion−electron interactions. The generalized gradient approximation (GGA) parametrized by Perdew, Burke, and Ernzerhof (PBE) was applied for the exchange-correlation functional[3, 4]. The standard PBE functional method was improved by Becke-Jonson damping potential with the DFT-D3 method [5], which is used for the empirical dispersion correction. The convergence criterion for the self-consistent iteration was 10^{-5} eV. The calculations were carried out by a plane-wave basis set cutoff energy of 400 eV and with a Gaussian smearing method of 0.05 eV, in order to assure well-converged total energy and force values. All positions and lattice parameters were fully relaxed and optimized to a force convergence of 0.001 eV $Å^{-1}$. The structural optimization of the hexagonal P6_3mc space group (186) of ZnO crystal structure was performed with the 5x5x3 Gamma-centered mesh method to generate k-points. The basis set cutoff energy was set to 520 eV with the Gaussian smearing method. The supercell was modeled as a periodically repeated slab of ZnO (002) phase of surface area 19.61×19.61 $Å^2$ extending along directions *x-y* and the *z*-direction separated perpendicularly with a vacuum space of 20.0 Å. The experimental cubic phase of $ZnIn_2S_4$ (Fd-3m) crystal structure with the lattice constants a=b=c is 10.622 Å. To test the reliability of our calculations, first examined by optimizing the bulk structure using the PBE with the DFT-D3 method.

Section II. Supporting Figures and Tables

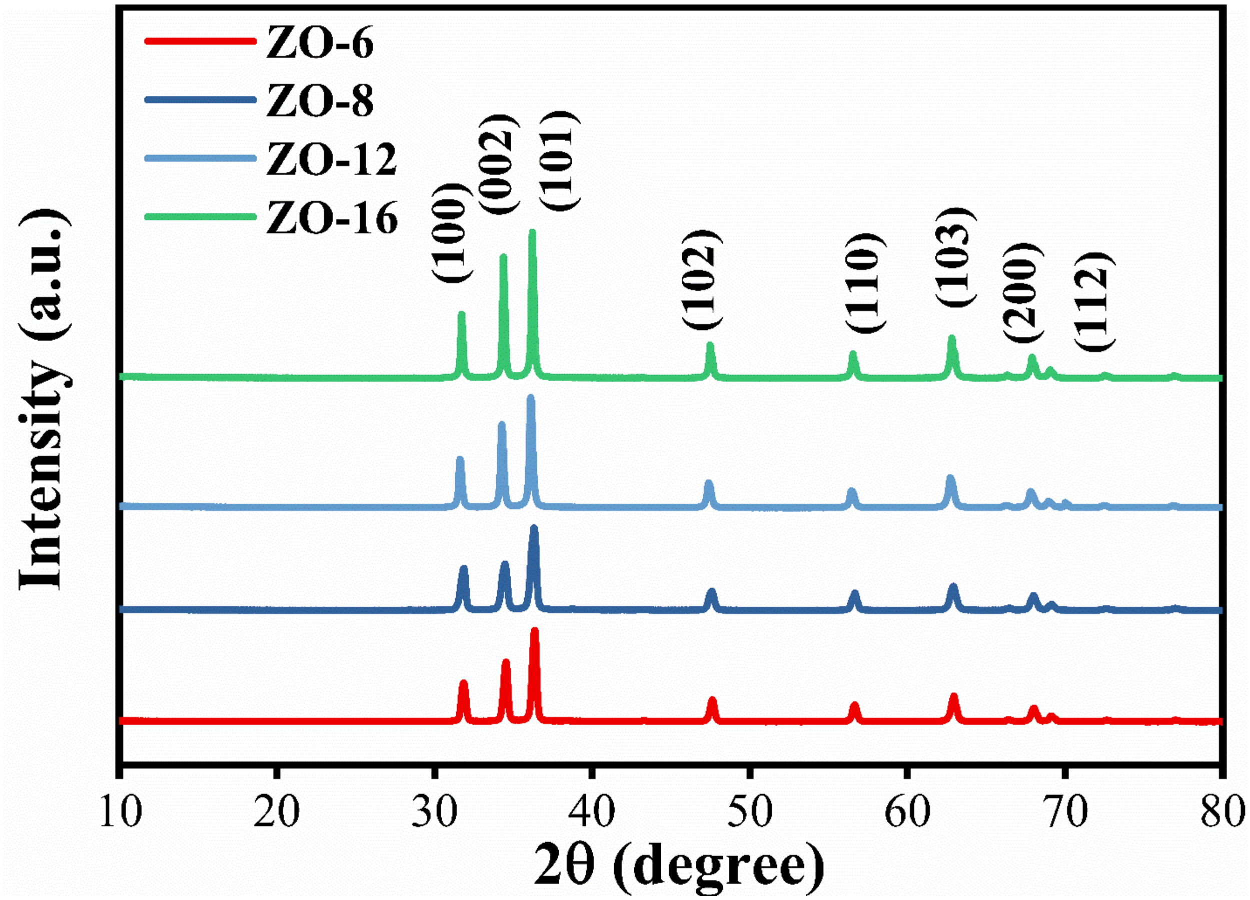

Figure S1. XRD patterns for ZO-x (x = 6, 8, 12, and 16h) prepared at different reaction times.

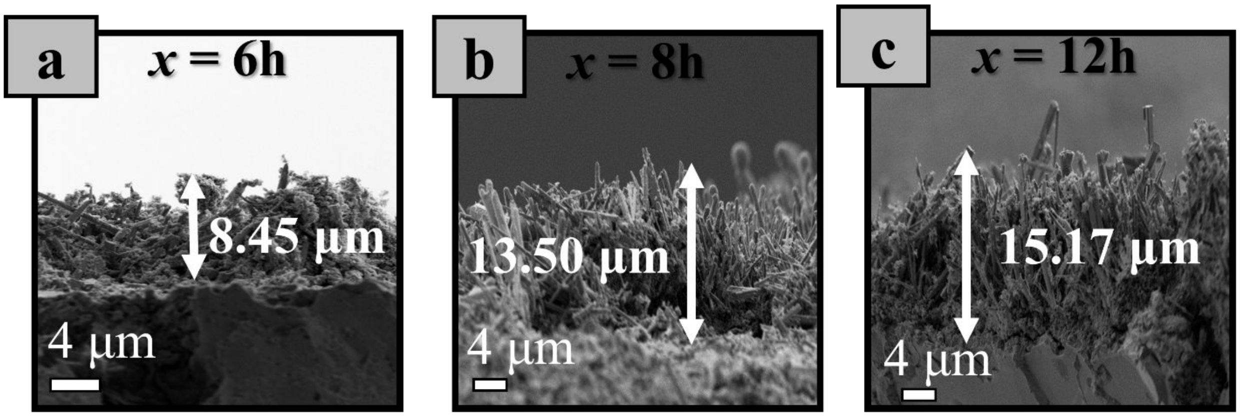

Figure S2. Cross-sectional FE-SEM images of (a) ZO-6, (b) ZO-8, and (c) ZO-12 catalysts.

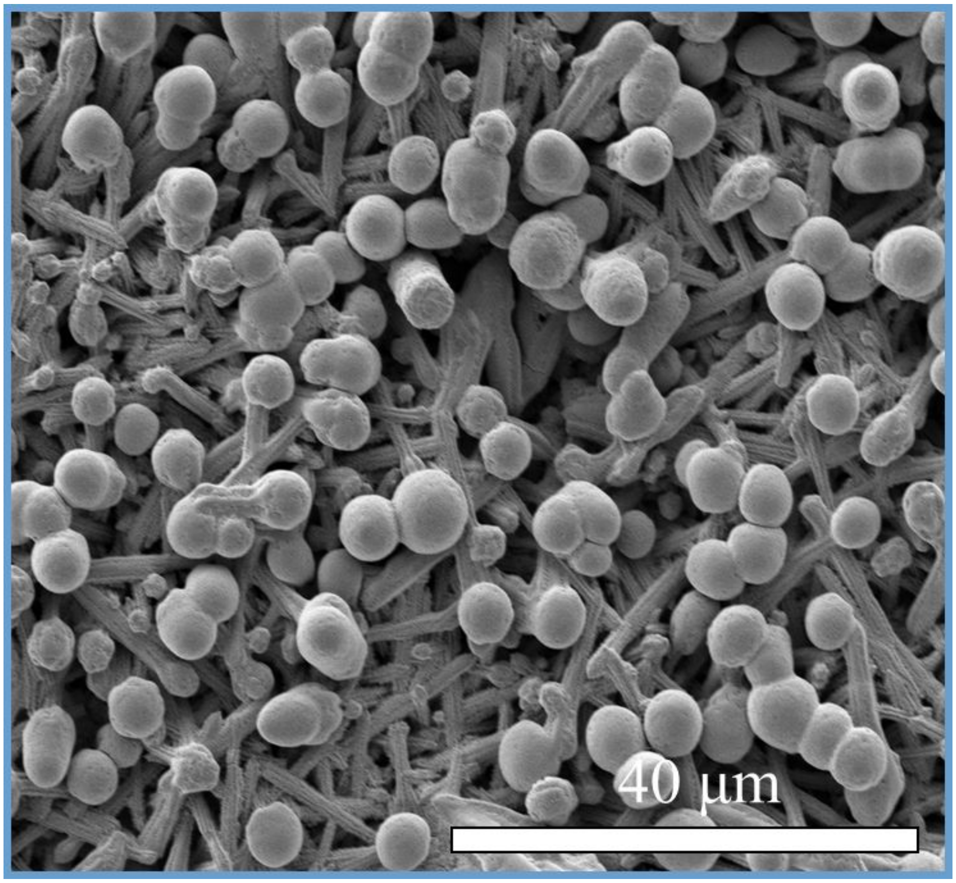

Figure S3. FE-SEM images for ZO-16 catalyst.

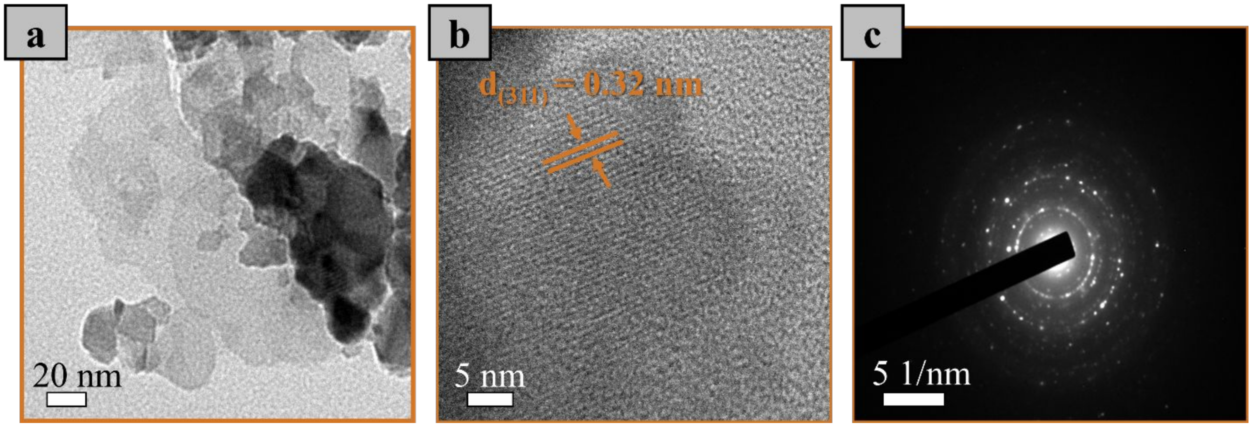

Figure S4. TEM (a), HR-TEM (b), and SAED pattern (c) images of ZIS material.

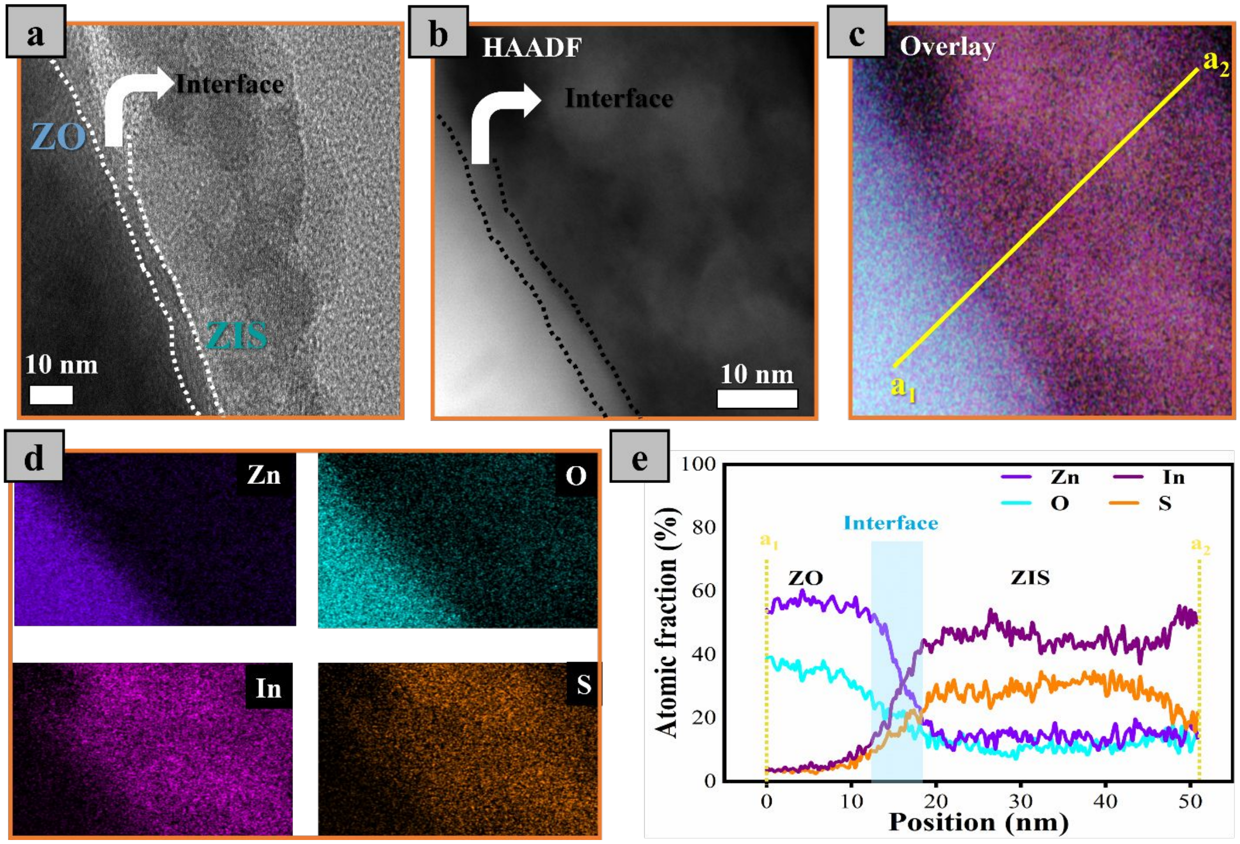

Figure S5. HR-TEM (a) and HAADF-STEM-EDX mapping (b-d) for ZIS/ZO-12 catalyst. (e) STEM-EDX line profile extracted from (c).

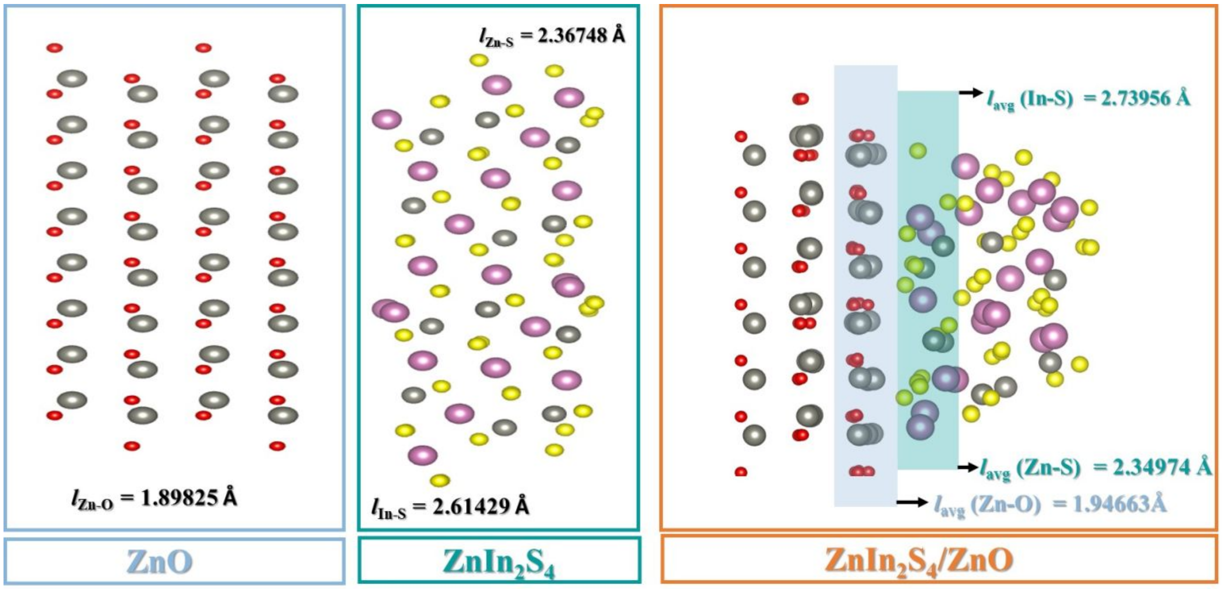

Figure S6. Simulated structures for ZnO, $ZnIn_2S_4$, and $ZnIn_2S_4$/ZnO catalysts.

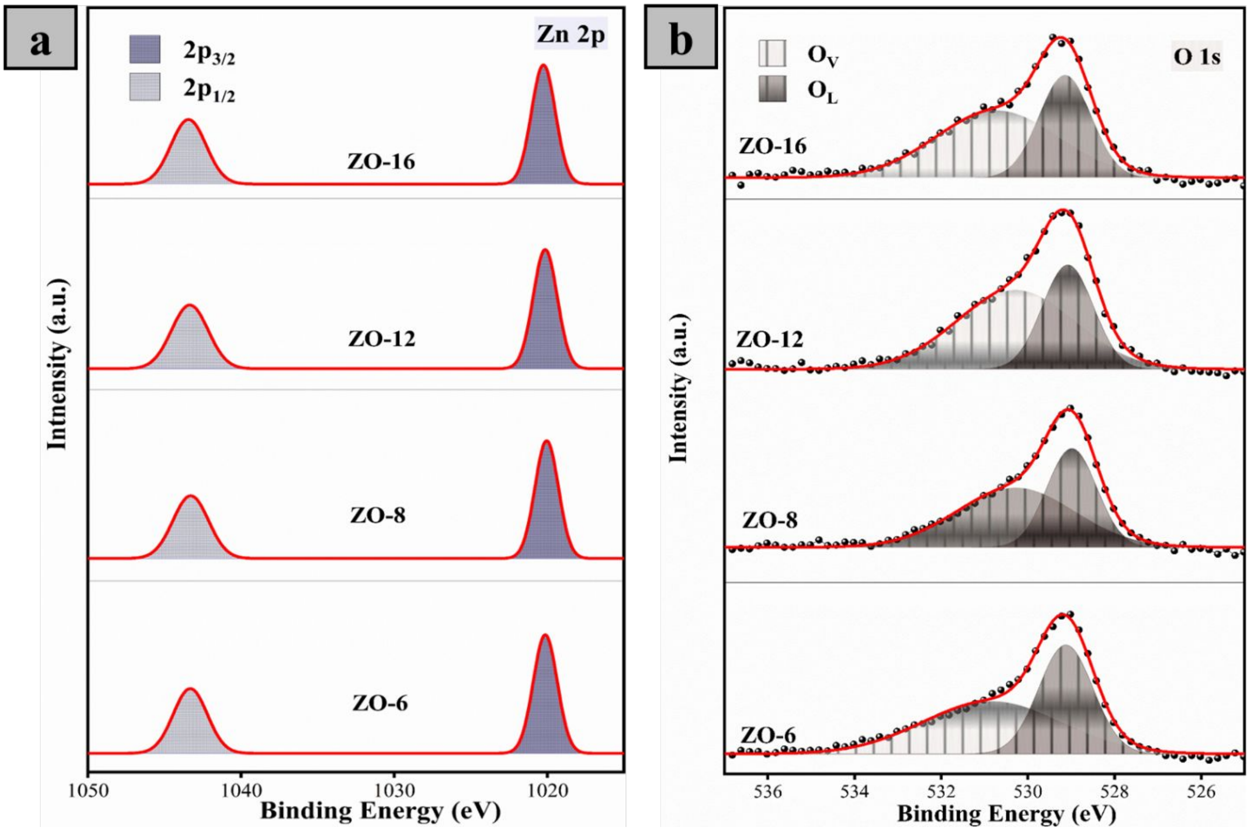

Figure S7. HR-XPS results of (a) Zn 2p and (b) O 1s for ZO-*x*

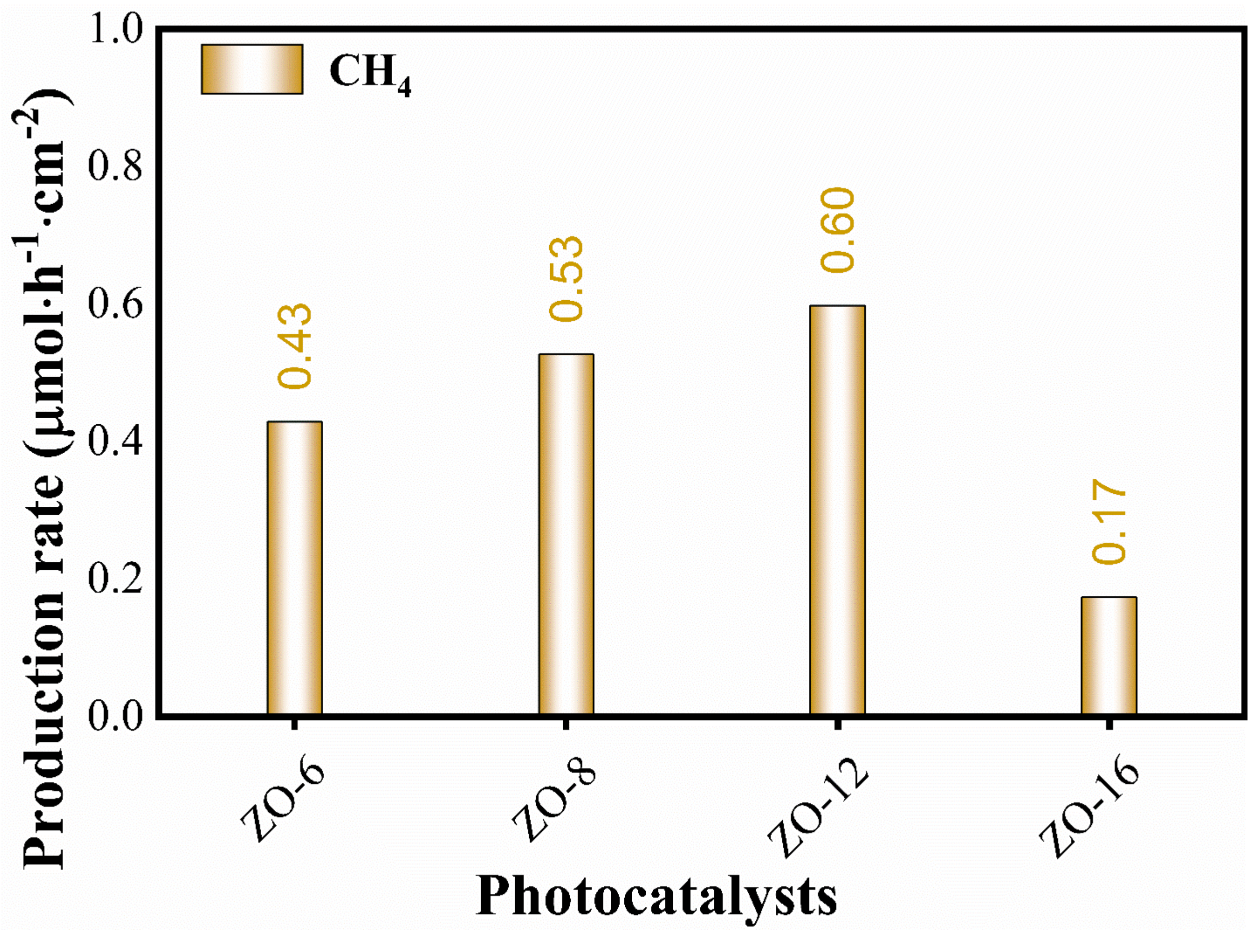

Figure S8. CH_4 Production rate measured by ZO-*x*.

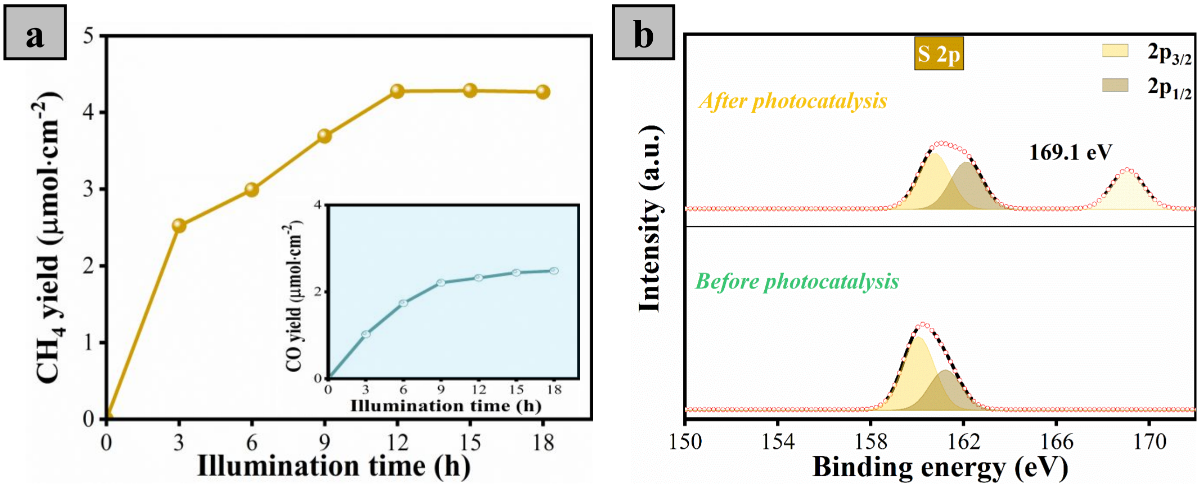

Figure S9. (a) CO and CH_4 Production yield measured by ZIS/ZO-12 as a function of radiation time. (b) HR-XPS spectra of S 2p for ZIS/ZO-12 before and after photocatalysis.

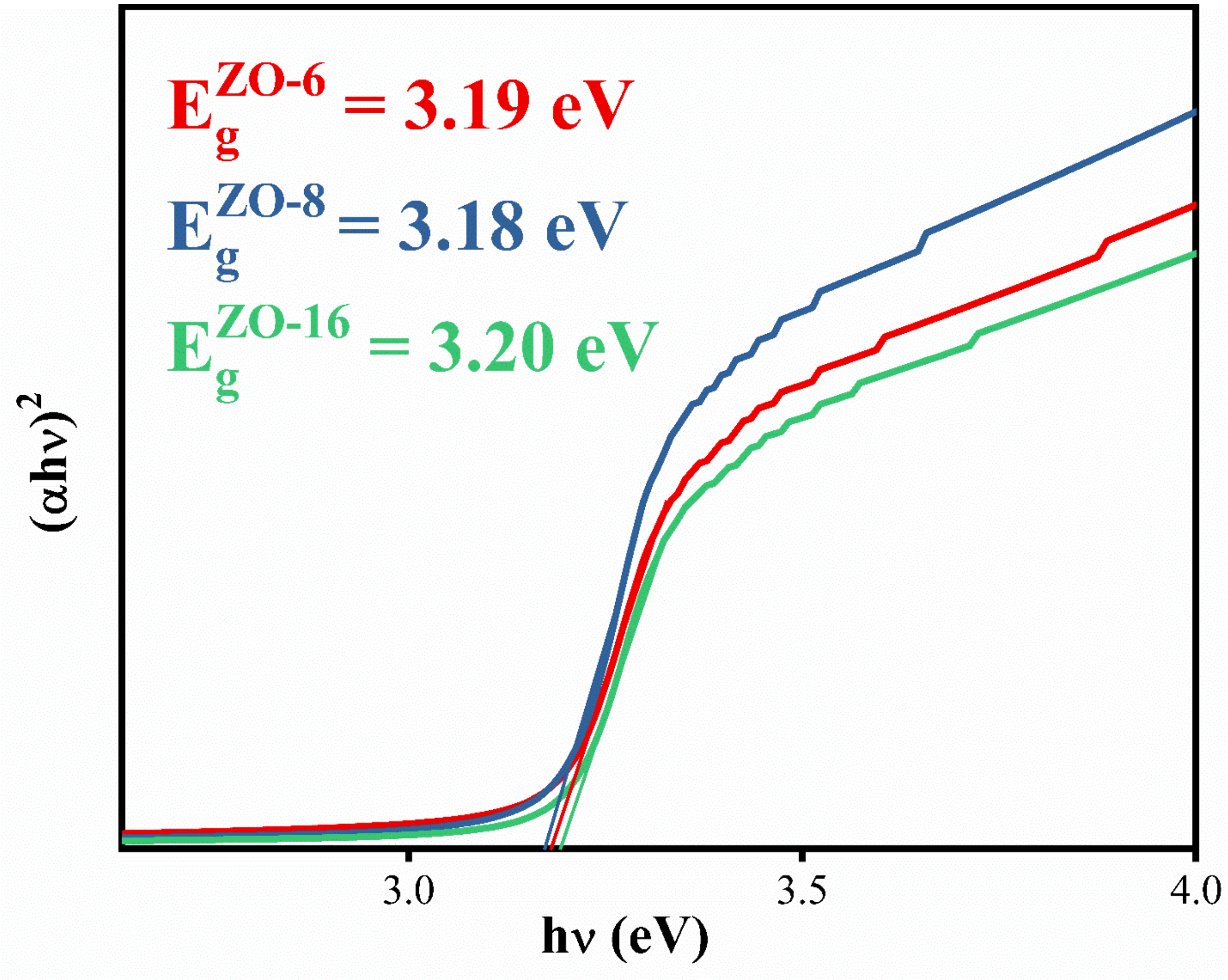

Figure S10. Tauc plots for ZO-6, ZO-8, and ZO-16 catalysts.

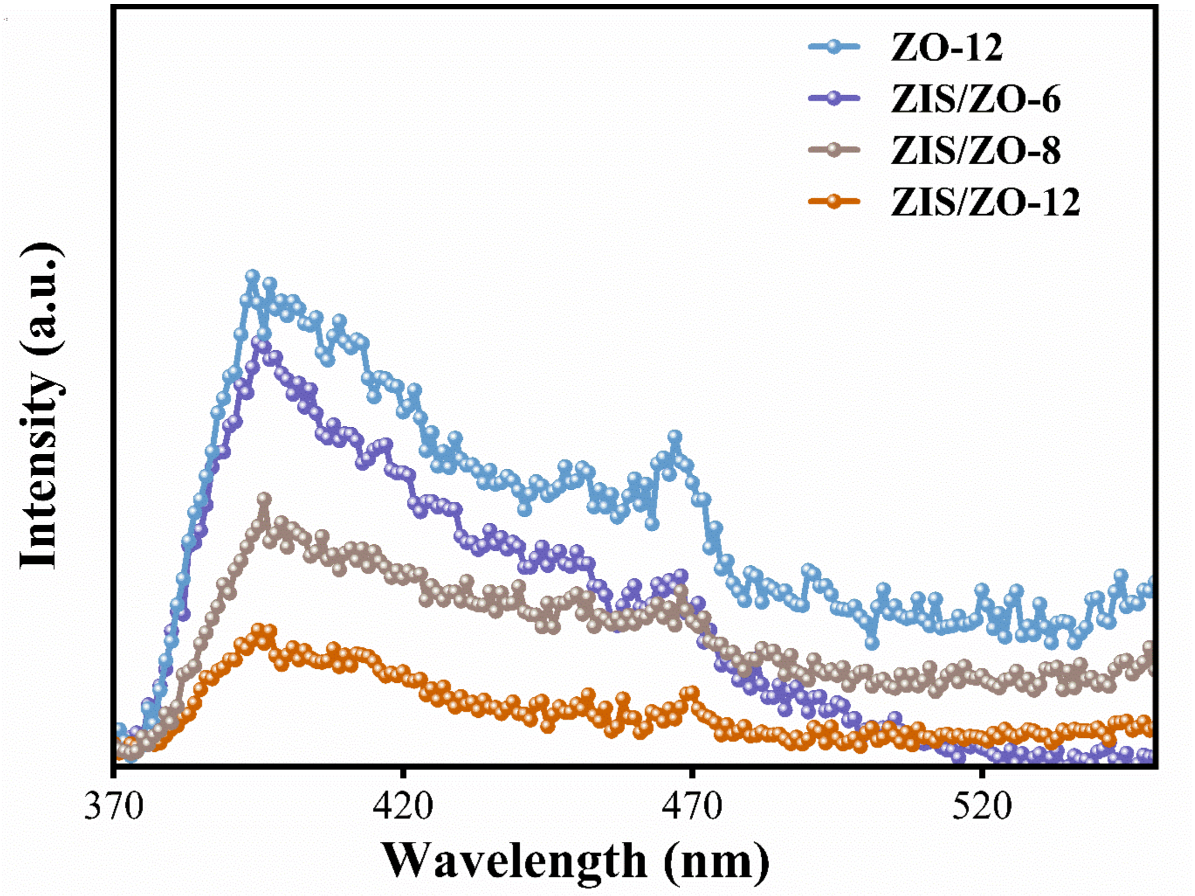

Figure S11. PL spectra for ZO-12, ZIS/ZO-6, ZIS/ZO-8, and ZIS/ZO-12 catalysts.

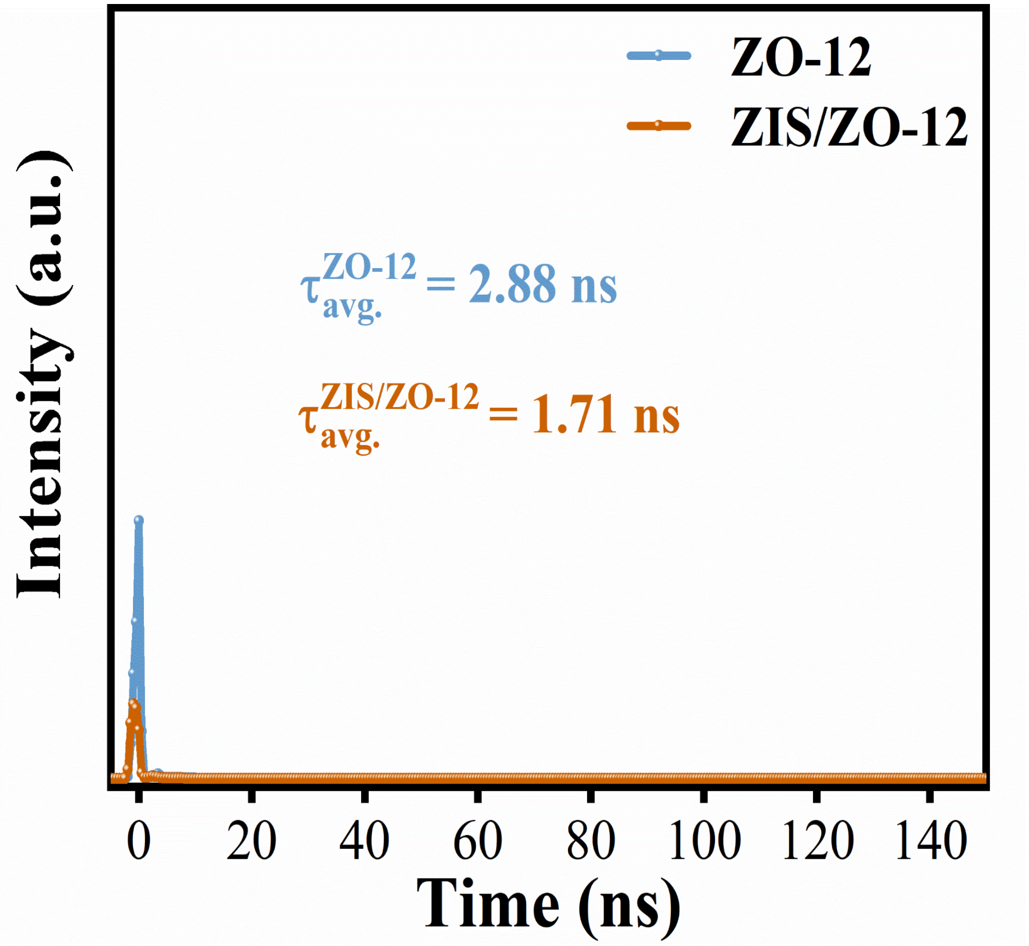

Figure S12. Time-resolved photoluminescence (TR-PL) spectra for ZO-12 and ZIS/ZO-12 catalysts.

Table S1. The peak area of oxygen vacancies (O_V) and lattice oxygen (O_L) from XPS results of ZO-*x* catalysts.

Catalyst	O_V	O_L
**ZO-6**	55.08%	44.92%
**ZO-8**	58.46%	41.53%
**ZO-12**	63.77%	36.23%
**ZO-16**	60.44%	39.56%
**ZIS/ZO-12**	60.73%	39.27%

Table S2. Comparison of CO_2 conversion efficiency by ZnO film-based photocatalysts.

Catalysts	Products	Production rate ($\mu mol \cdot cm^{-2} \cdot h^{-1}$)	Reference
**$ZnIn_2S_4$/ZnO**	CO	0.34	This work
	CH_4	0.84	
**ZnO/ZnTe**	CH_4	0.04	[6]
**ZnO/Au/g-C_3N_4**	CO	0.0086	[7]
**ZnO/Au**	CO	0.0044	[7]
**ZnO photonic crystal**	CO	~ 0.071	[8]

REFERENCES

1. Lee, C.; Yang, W.; Parr, R. G., Development of the Colle-Salvetti correlation-energy formula into a functional of the electron density. *Physical review B* **1988,** *37* (2), 785.
2. Perdew, J. P.; Burke, K.; Ernzerhof, M., Generalized gradient approximation made simple. *Phys. Rev. Lett.* **1996,** *77* (18), 3865.
3. Kresse, G.; Furthmüller, J., Efficient iterative schemes for ab initio total-energy calculations using a plane-wave basis set. *Physical review B* **1996,** *54* (16), 11169.
4. Kresse, G.; Hafner, J., Ab initio molecular dynamics for liquid metals. *Physical review B* **1993,** *47* (1), 558.
5. Tkatchenko, A.; DiStasio Jr, R. A.; Car, R.; Scheffler, M., Accurate and efficient method for many-body van der Waals interactions. *Phys. Rev. Lett.* **2012,** *108* (23), 236402.
6. Iqbal, M.; Wang, Y.; Hu, H.; He, M.; Shah, A. H.; Li, P.; Lin, L.; Woldu, A. R.; He, T., Interfacial charge kinetics of ZnO/ZnTe heterostructured nanorod arrays for CO2 photoreduction. *Electrochim. Acta* **2018,** *272*, 203-211.
7. Li, X.; Jiang, H.; Ma, C.; Zhu, Z.; Song, X.; Wang, H.; Huo, P.; Li, X., Local surface plasma resonance effect enhanced Z-scheme ZnO/Au/g-C_3N_4 film photocatalyst for reduction of CO_2 to CO. *Appl. Catal. B .* **2021,** *283*, 119638.
8. Wu, X.; Lan, D.; Zhang, R.; Pang, F.; Ge, J., Fabrication of opaline ZnO photonic crystal film and its slow-photon effect on photoreduction of carbon dioxide. *Langmuir* **2018,** *35* (1), 194-202.
